# O01 Nuances of implementing antibiotic stewardship in high-prescribing English general practices: an implementation study

**DOI:** 10.1093/jacamr/dlac052.001

**Published:** 2022-05-31

**Authors:** Sarah Tonkin-Crine, Monsey McLeod, Aleksandra Borek, Anne Campbell, Phillip Anyanwu, Ceire Costelloe, Mike Moore, Benedict Hayhoe, Chris C Butler, Alison Holmes, A Sarah Walker

**Affiliations:** 1 University of Oxford, Oxford, UK; 2 NHS England, UK; 3 Imperial College London, London, UK; 4 Cardiff University, Cardiff, UK; 5 The Institute of Cancer Research, UK; 6 University of Southampton, Southampton, UK

## Abstract

**Background:**

Trials have identified several antimicrobial stewardship (AMS) strategies that effectively reduce antibiotic use in primary care. However, many are not commonly used in England and evidence is lacking around how to support high-prescribing general practices to implement AMS. We co-developed a customizable implementation intervention to improve adoption and use of three AMS strategies; enhanced communication strategies with/without patient leaflets, delayed prescriptions, and point-of-care C-reactive protein testing (POC-CRPT). This study aimed to investigate the use of the implementation intervention in high-prescribing practices and its effect on antibiotic prescribing.

**Methods:**

Nine high-prescribing practices had access to the intervention for 12 months from November 2019. This was primarily delivered remotely via the ‘Antibiotic Optimisation’ website with practices required to identify an in-practice ‘Antibiotic Champion’. In a mixed-methods evaluation, we compared routinely collected prescribing data between 9 intervention and 45 matched control practices using a difference in differences analysis. Intervention material use was assessed through website monitoring and practice requests for supplies. Surveys and interviews were conducted with professionals at baseline and follow-up timepoints to capture experiences of using the intervention.

**Results:**

There was no evidence that the intervention affected antibiotic prescribing. Engagement with intervention materials differed substantially between practices and depended on the individual Antibiotic Champion's preconceptions regarding the AMS strategies and opportunity to do the implementation tasks. Champions in five practices initiated changes to encourage use of at least one of the three AMS strategies, mostly POC-CRPT and patient leaflets. One Champion used the website resources to drive discussions about implementation of all three strategies in their practice. Elsewhere, lack of website engagement meant clinicians did not recognize novelty in the communication and delayed prescription content. POC-CRPT was used most often when equipment was allocated to one person in a practice.

**Conclusions:**

AMS strategies are complex interventions and require clinicians to have detailed knowledge on how to adopt them in practice if they are to reduce antibiotic prescribing. Successful adoption may be achieved by allocating one or two persons to use an AMS strategy rather than all clinicians. Although effective in trials, remote, one-sided provision of AMS strategies is unlikely to change prescribing; initial clinician engagement and understanding needs to be monitored to avoid misunderstanding and suboptimal use.

